# Chiral liquid crystal elastomers advance light modulation

**DOI:** 10.1038/s41377-024-01549-4

**Published:** 2024-08-23

**Authors:** Jiazhe Ma, Zhongqiang Yang

**Affiliations:** 1 Department of Chemistry, Key Lab of Organic Optoelectronics and Molecular Engineering of Ministry of Education, Tsinghua University, Beijing, 100084 China; 2 Laboratory of Flexible Electronics Technology, Tsinghua University, Beijing, 100084 China

**Keywords:** Optical materials and structures, Electronics, photonics and device physics

## Abstract

Chiral liquid crystal elastomers, as soft photonic materials, enable dynamic omnidirectional tuning of circularly polarized reflection wavelength and function as an effective medium for full-color circularly polarized luminescence, showing promise for advanced photonic applications.

Chiral liquid crystal elastomers have emerged as a distinctive class of photonic materials that exhibit both the anisotropic order of liquid crystals and the elasticity of rubbers. As representatives, cholesteric liquid crystal elastomers (CLCEs) possess a one-dimensional periodic helical molecular arrangement^1^, whereas blue-phase liquid crystal elastomers (BPLCEs) exhibit a double-twist cylinder intertwined with a three-dimensional lattice of adjacent disclinations^2,3^. These unique chiral nanostructures, coupled with elastomeric frameworks, not only enable them to selectively reflect circularly polarized light but also confer them with exceptional optical modulation capabilities, making them widely applicable in adaptive optics^4^, information storage^5^, and so on.

A notable feature of chiral liquid crystal elastomers is their mechanochromic behavior^6^. For instance, in CLCEs, external forces trigger color changes across a broadband wavelength range by modifying the helical pitch length. This property renders CLCEs promising materials for tunable structural colors in photonic applications such as stress sensors^7^ and bioinspired camouflage^8^. However, most existing research on wavelength tuning has been limited to unidirectional shifts toward shorter wavelengths, primarily due to the pitch-contractive helix reconfiguration induced by the stretching deformation of CLCEs, resulting in a reduction of the optical period. This limitation restricts their tuning range and on-demand wavelength tunability.

To achieve both broadband and completely unrestricted wavelength control, a recently published study in Light: Science & Applications by Su Seok Choi et al. from the Department of Electrical Engineering at Pohang University of Science and Technology (POSTECH), Korea, reported the omnidirectional color wavelength tuning of CLCEs toward longer and shorter wavelengths over a broadband wavelength range^9^. As depicted in Fig. 1, a novel methodology involving a bi-mode dielectric elastomer actuator (DEA) system was designed to achieve omnidirectional wavelength tuning of CLCEs. Specifically, the DEA incorporates two distinct electrode configurations: a circular-shaped compliant electrode for inducing positive strain and a donut-shaped compliant electrode for inducing negative strain. Upon applying an electric voltage, the DEA operates in two modes. In the stretching mode, the circular electrode expands, leading to a positive strain in the CLCE, which results in a reduction of the helical pitch length and a shift toward shorter wavelengths. Conversely, in the contraction mode, the donut-shaped electrode contracts, inducing negative strain in the CLCE, which causes an expansion of the helical pitch length and a shift toward longer wavelengths. This bi-modal operation of the DEA allows for simultaneous and omnidirectional structural color tuning of CLCEs by generating both expansion and contraction deformations. The negative strain effect achieved through the contraction mode DEA is particularly noteworthy as it enables tuning of CLCEs toward longer wavelengths, overcoming the limitations of previous studies focused solely on shorter wavelength tuning. With on-demand omnidirectional wavelength control and a broad tuning range of approximately 190 nm, this work enhances the circularly polarized reflection capabilities of CLCEs, paving the way for innovative applications in photonic devices.

**Fig. 1:**
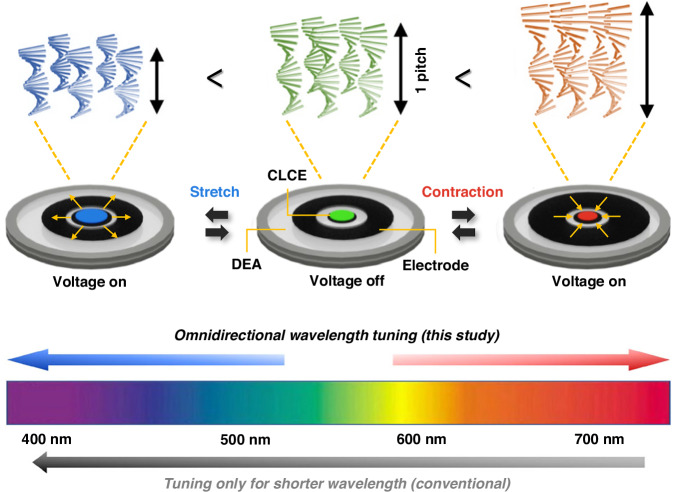
Concept and design of omnidirectional wavelength tuning of CLCEs by a DEA system. The system features two electrode modes: circular for stretching and donut-shaped for contraction, allowing for both pitch-contraction and pitch-expansion in CLCEs, and enabling color tuning towards shorter and longer wavelengths, respectively

Beyond the remarkable ability to modulate circularly polarized reflection, chiral liquid crystal elastomers, owing to their unique chiral structures, also serve as ideal soft templates for circularly polarized luminescence (CPL), which holds potential applications in various fields^10^ such as biosensing, three-dimensional displays, and information encryption. CPL refers to the differential emission of left- and right-handed circularly polarized light, and the luminescence dissymmetry factor (*g*_lum_) is a crucial parameter for evaluating CPL systems. While using CLCEs with helical superstructures has proven effective in amplifying the *g*_lum_ value^11^, achieving magnified *g*_lum_ values in CLCEs typically requires precise modulation of the chiral agent content to match the emission and reflection bands of the system.

To address the challenges in adequately and precisely tuning CPL in polymer-based materials, in a new paper published in Light: Science & Application, a team led by Professor Jinbao Guo from the College of Materials Science and Engineering, Beijing University of Chemical Technology, and Professor Quan Li from the Institute of Advanced Materials and School of Chemistry and Chemical Engineering, Southeast University, China, has reported a breakthrough in utilizing BPLCEs doped with quantum dots (QDs) to achieve visualized full-color and mechanically-switchable CPL (Fig. 2)^12^. Despite similar circular dichroism signals, the CPL signal of BPLCEs exhibits opposite chirality compared to that of CLCEs, highlighting their distinct mechanisms. While CLCEs selectively reflect right-handed circularly polarized light and transmit left-handed circularly polarized light, resulting in a left-handed CPL signal, the CPL of BPLCEs is induced by their highly ordered 3D structure and chiral environment, consistently yielding a right-handed CPL signal, regardless of the matching between photonic bandgaps (PBGs) and emission bands of QDs. This approach achieves visualized full-color CPL with a maximum *g*_lum_ absolute value of up to 0.74 by doping red, green, and blue QDs emitters, respectively. Furthermore, the inherent elastomeric properties of BPLCEs enable toggling the CPL signal on and off by longitudinally extending the 3D lattice via mechanical stretching, and the extinction of CPL signals can be permanently fixed via dynamic disulfide bonds within the BPLCEs. As proof of concept, the potential applications of BPLCEs-based CPL materials in anti-counterfeiting and information encryption were demonstrated. The primary attraction of this work lies in breaking the limitation posed by the reliance of magnified *g*_lum_ values in CLCEs on the match between PBGs and emission bands, thus facilitating a more facile and effective modulation of CPL. Additionally, this innovation broadens the application scope of CPL materials and provides a new perspective for the design of advanced optoelectronic devices.

**Fig. 2:**
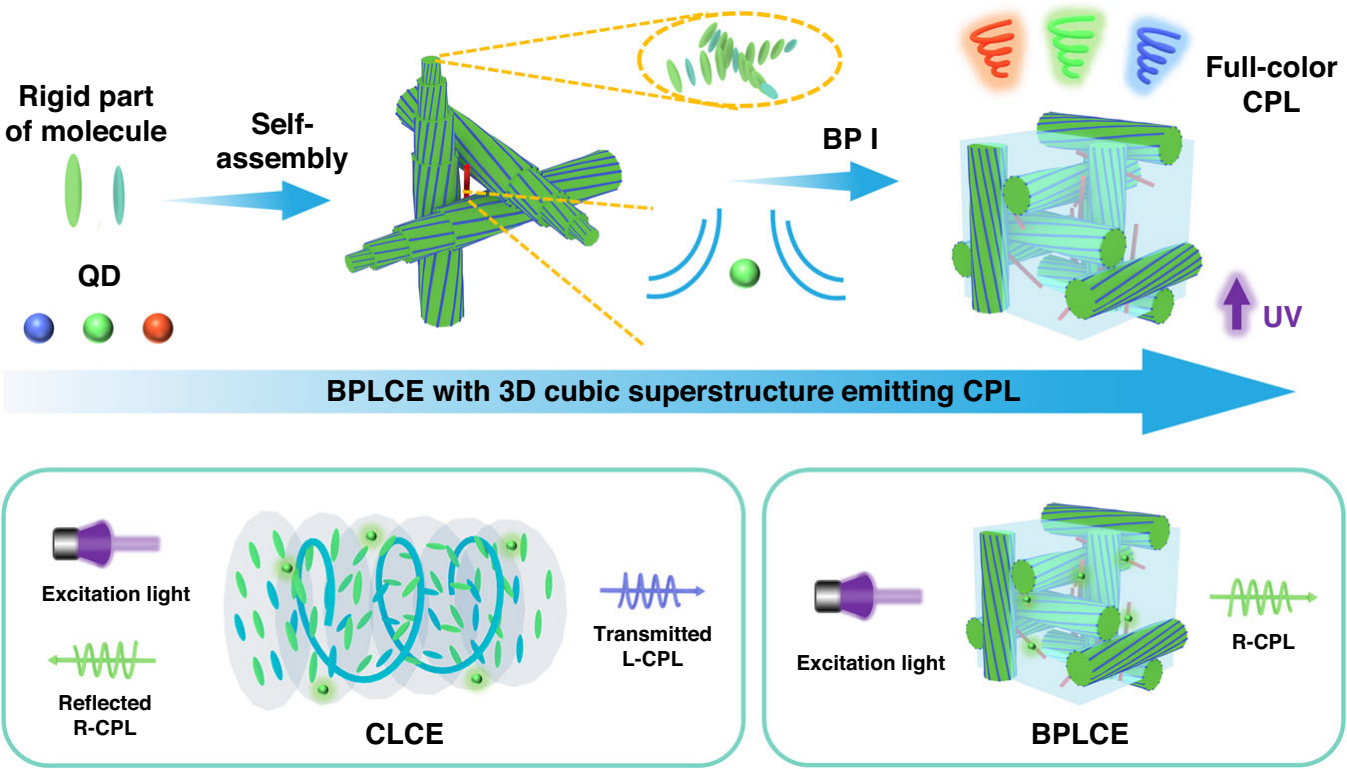
Schematic diagram of CPL signals emission from BPLCEs. Visualized full-color CPL is achieved by doping BPLCEs with red, green, and blue QD emitters, respectively. Unlike the selective reflection mechanism in CLCEs, which produces left-handed CPL signals, BPLCEs exhibit right-handed CPL signals due to their unique 3D cubic superstructure and chiral environment

In summary, chiral liquid crystal elastomers, particularly those featuring cholesteric and blue-phase structures, constitute a promising class of materials due to their versatile optomechanical properties. Their light modulation capabilities on omnidirectionally tunable structural color wavelength and visualized full-color and mechanically-switchable CPL, coupled with their potential applications in areas such as adaptive photonics, bioinspired devices, and security systems, underscore their significance for advancing cutting-edge technologies and opening up new avenues for research and development.
